# An Integrated Cognitive-Motivational Model of Ikigai (Purpose in Life) in the Workplace

**DOI:** 10.5964/ejop.9943

**Published:** 2023-11-30

**Authors:** Mégane Sartore, Stéphanie Buisine, Ioana Ocnarescu, Louis-Romain Joly

**Affiliations:** 1 SNCF, Research Direction, Paris, France; 2 CESI-LINEACT, CESI, Paris, France; 3 Strate Research, Strate School of Design, Paris, France; Victoria University of Wellington, Wellington, New Zealand

**Keywords:** ikigai, work motivation, well-being, mindfulness

## Abstract

In the Japanese philosophy of life, ‘ikigai’ broadly refers to having a ‘reason for living’, or a purpose in life. From a phenomenological and empirical viewpoint, ikigai is reported to increase human well-being and even life expectancy. However, it remains difficult to translate, define and formalize with regard to contemporary psychological theories. In this respect, the aim of this paper is twofold: to capture as accurately as possible what ikigai is, and to examine whether the concept applies to a professional context. We first offer a comprehensive overview of ikigai, bridge the gap between this specific body of literature and related psychological theoretical frameworks, such as those addressing motivation, well-being, and attention. On this basis, we conceptualize an integrated cognitive-motivational model of ikigai using an IPO (Input-Process-Output) framework: we organize dispositional or situational factors supposedly supporting ikigai as inputs, fueling the core process of ikigai (mainly built from motivational and attentional mechanisms), which produce outcomes (including well-being). A feedback loop completes the model and allows the process to maintain over time. This conceptual proposal is a first step towards applying and testing the model in professional contexts, in order to renew our approach of engagement, well-being, and performance at work as well as inspire workplace evolution.

Ikigai was introduced in Japanese literature by Kamiya (1966) and, although it has no exact translation, it refers to a sense of “life worth living” (Kotera et al., 2021; Weiss et al., 2005), encompassing well-being (Shirai et al., 2006), “purpose in life” or “reason for living” (Mathews, 1996; Mori et al., 2017; Sone et al., 2008). It can be defined as “a feeling obtained by a person who is doing something useful for someone else or society and, consequently, feels that life is worth living” (Fukuzawa et al., 2019, p. 1). However, to date, there is no consensual academic definition of ikigai (Kumano, 2003).

Two decades of essentially Japanese empirical research on ikigai have been conducted in medicine (Ishida, 2012; Nakanishi, 1999; Shirai et al., 2006; Sone et al., 2008), psychology (Fukuzawa et al., 2019; Kamiya, 1966; Kumano, 2012, 2018), education (Hikmawan et al., 2019), anthropology (Mathews, 1996; Murray, 1993), and social sciences (Kono et al., 2019). Ikigai is said to improve health (Nakanishi, 1999) and longevity (Sone et al., 2008; Tanno et al., 2009) by reducing risks of all-cause mortality. As such, it appears as an inspiring concept intrinsically linked to Japanese unique culture.

Our aim is first to understand whether ikigai can be fully modelled based on existing psychological theories, or whether it brings a new approach to scholarly view on well-being, motivation and related concepts. After presenting literature dedicated to ikigai in the first place, we will link it to current psychological theories, including Self-Determination Theory (Deci & Ryan, 2000), the PERMA theory of building blocks of well-being (Positive emotions, Engagement, positive Relationships, Meaningfulness and Accomplishment; Seligman, 2011), and Mindfulness (Ryan et al., 2008). Secondly, we also wish to bridge the gap between ikigai as a philosophy of life and self-fulfillment at work, which can be approached for example through the Theory of Purposeful Work Behavior (Barrick et al., 2013), or the Job Characteristics Model (Hackman & Oldham, 1976). We present these insights organized as a process that we call the integrated cognitive-motivational model of ikigai.

## Ikigai as a Unique Japanese Concept

Beyond linguistic specificities, ikigai may be difficult to translate and define because of cultural specificities. Anthropological studies have emphasized differences between Japanese and North American self (Kotera et al., 2021; Mathews, 1996; Smith, 1991). Japanese self may be more contextual and socio-centric (Mathews, 1996), with Japan’s cultural profile lying in the middle (46/100) of the individualism/collectivism dimension (Hofstede et al., 2010). In contrast, North American conception of the self seems particularly individualistic (Spiro, 1993), specifically in the USA (which scores 91/100 on individualism; Hofstede et al., 2010). In individualistic cultures *“societies exist to promote the well-being of individuals”* (Oyserman & Lee, 2008, p. 311); individuals are encouraged to define themselves as autonomous and distinct from others. Conversely, collectivism is group-centered: *“societies exist, and individuals must fit into them”* (Oyserman & Lee, 2008, p. 311), which means that individuals are encouraged to define themselves in terms of relationships with others. Consistently, East Asian people value interdependence (to friends and family), whereas North Americans value independence (Fukuzawa et al., 2019; Markus & Kitayama, 1991). Individualistic cultures are also more horizontal (equality of relationships with others), while collectivist cultures are more vertical (hierarchy; Singelis et al., 1995). Finally, people from individualistic and collectivistic cultures have different cognitive patterns and values which affect the way they interpret information and make decisions (Oyserman & Lee, 2008): cultures may value intrinsic or extrinsic motivation, and influence how universal needs are expected to be met. For example, cultures have different definitions of achievement: collectivist cultures value contribution to the group while individualist cultures value individual accomplishment (Ryan & Deci, 2000). Similarly, individualistic cultures focus on personal needs and desires, while collectivistic cultures consider the needs and desires of others (Markus & Kitayama, 1991). In this context, how would people from individualistic cultures capture the concept of ikigai?

**Figure 1:**
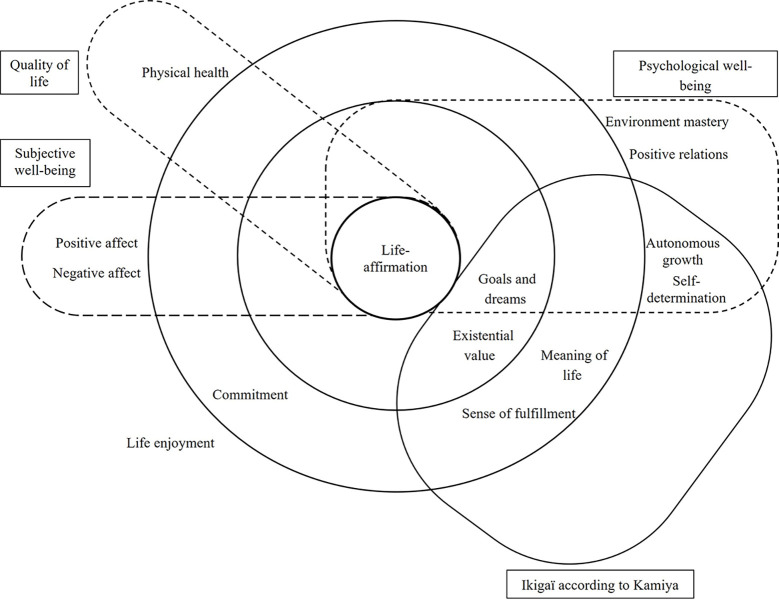
The Structure of Ikigai and Similar Concepts *Note.* Adapted from Kumano, 2006, our translation.

In Japan, the desire for ikigai is considered universal (Kamiya, 1966; Nakanishi, 1999) and may contribute to meet seven needs: (1) Survival, (2) Growth and Change, (3) Future such as life goals and dreams, (4) Influence (being necessary to others), (5) Freedom of choice, (6) Self-fulfilment or personal development through one's potential (autonomous growth), and (7) Meaning of life (a sense of value and worth of one's own life). In an attempt to formalize ikigai (Kumano, 2006, 2012), four factors described as psychological states were identified (Life-affirmation, Meaning of life, Life fulfilment, and Existential value), as well as five cognitive value-laden mechanisms through which people perceive life worthiness: (1) making sense of the past, (2) setting future goals, (3) being absorbed in the positive present, (4) accepting negative situations, and (5) coping with negative situations. Kumano (2013) further emphasizes the link between the four-factor model and the five value-laden mechanisms. This conceptualization gave rise to a hierarchical model highlighting central and peripheral elements of ikigai (Figure 1, Kumano, 2006, 2012). Key components of ikigai would be life-affirmation, goals and dreams, meaning of life, existential value, a sense of fulfilment, and commitment. Subjective well-being, psychological well-being, and quality of life would not be central to ikigai (Kumano, 2012).

This model provides a better understanding of the Japanese view of ikigai. It also highlights a few inconsistencies between Kumano's and Kamiya's views regarding the core concepts of ikigai (as pointed out in Figure 1). Finally, although this model has been used in many Japanese ikigai studies, it lacks parsimony.

In Ohsaki's study (Sone et al., 2008), which is a longitudinal study with 43,391 participants over seven years, ikigai was measured through simple questions like “Do you have ikigai in your life?” (Sone et al., 2008; Tanno et al., 2009). The results suggested that subjects who did not find their ikigai exhibited higher risks of all-cause mortality. Ikigai was mainly investigated with elderly people to study longevity in blue zones^1^1The five blue zones are regions of the world where a significant number of people live much longer and better. They are Ikaria in Greece, Okinawa in Japan, Sardinia in Italia, Loma Linda in USA and Nicoya Peninsula in Costa Rica. (Fukuzawa et al., 2019; Nakanishi, 1999; Shirai et al., 2006; Tanno et al., 2009), and with students (Hikmawan et al., 2019; Kono et al., 2019; Kumano, 2003). These studies highlight, for example, the role of social network for elderly people (Fukuzawa et al., 2019), and ikigai decline with age (Fukuzawa et al., 2019; Nakanishi, 1999).

A large part of these studies was conducted in Japan (Fukuzawa et al., 2019; Iida & Oguma, 2013; Kono et al., 2019; Shirai et al., 2006; Sone et al., 2008; Tanno et al., 2009), others in Indonesia (Hikmawan et al., 2019). In Europe, Ikigai-9 scale (Imai, 2012) was translated into English and French (Fido et al., 2020; Vandroux & Auzoult-Chagnault, in press) but did not give rise to intercultural studies to date. Some studies published only in Japanese (Kumano, 2003, 2006) support the cultural specificity of the concept (Nakanishi, 1999), which may question the portability of this life philosophy to other cultures.

**Figure 2:**
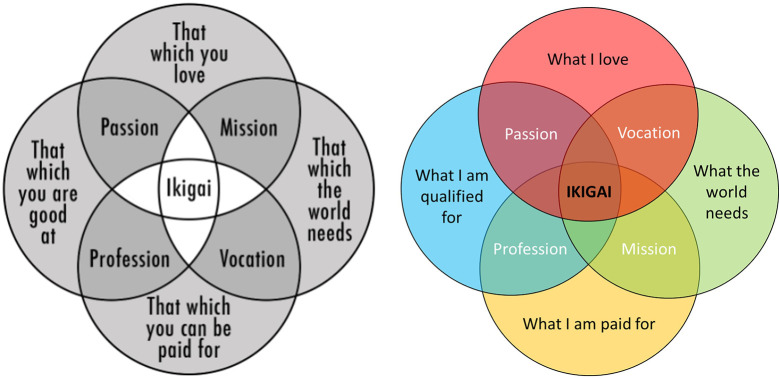
Diagram of Ikigai (Left) and Our Adaptation (Right) *Note*. Diagram of ikigai from Winn (2014).

In the USA, ikigai is represented by Winn's diagram (Figure 2), which builds on four areas: “what you love”, “what the world needs”, “what you are good at”, and “what you are paid for”, the intersection of which being named ikigai. This diagram does not come from the scientific literature and its origins are uncertain. Initially, it was meant to represent purpose (Zuzunaga, 2012): the “Purpose Venn Diagram” ultimate intersection was named “purpose” (i.e., “Propósito” in Spanish) instead of ikigai. Besides, a TedTalk conference titled “How to live 100+” (Buettner, 2009) revealed to a predominantly North American audience that ikigai was a reason for long-living in Okinawa. These two sources may have inspired the publication of a blog post (Winn, 2014) presenting an adaptation of the Purpose Venn Diagram renaming the intersection “ikigai”. Winn (2014) also renamed the field “what you are paid for” by “that which you can be paid for” without elaborating on his choice, while the other three areas are formulated as achievements, not potentials. Intermediary intersections also show discrepancies between “profession” and “mission”. We present below an iteration on Winn's (2014) diagram with simplified labels for the areas (Figure 2) and use of the first person for appropriation purposes. We also swapped “mission” and “vocation”, as a “mission” refers to an important assignment and may be more likely associated to an external reward while “vocation” refers to a strong sense of fit for a career and may be more likely associated to personal liking.

The viral nature of Winn's (2014) diagram could be due to its simplicity and accessibility to represent a profound Japanese concept. It is also inspiring as it prompts anyone to question life meaningfulness. Another strength of this representation is to articulate personal factors (“what I love” and “what I am qualified for”), external rewards (“what I am paid for”) and altruistic purposes (“what the world needs”). Indeed, ikigai is positively correlated to a cooperative view of life and negatively correlated to contemplative life (Kumano, 2003). These features emphasize the significance of interpersonal or outward achievement in building a sense of self.

This diagram nonetheless shows some limitations. For example, the economic dimension (“what you are paid for”) is subject to debate as ikigai can be reached independently from any financial reward (Nakanishi, 1999). Ikigai can be both the source of value in one's life or what makes life worth living and the mental and spiritual circumstances under which individuals feel that their lives are valuable. Beyond work, ikigai can be reached in one's family life or leisure time. Economic rewards are usually not referred to in Japanese literature except for one research including financial status as social capital (Fukuzawa et al., 2019).

Given these issues, a theoretical and testable model of ikigai is needed for improving our understanding of this concept and inspire new ways of viewing life and work. To our best knowledge, no attempt to provide a cognitive conceptualization of ikigai was made before.

## An Integrated Cognitive-Motivational Model of Ikigai

The lack of formal model of ikigai makes it difficult to use it as an intervention framework in the field. This is consistent with the view that ikigai is a complex process and its role in mental and physical conditions difficult to measure" (Nakanishi, 1999, p. 323). Our model highlights both preconditions and benefits in terms of well-being (Shirai et al., 2006), health (Nakanishi, 1999; Sone et al., 2008), and performance (our hypothesis).

A cognitive process can be described through a causality chain linking Inputs, core Processes, and Outputs (I-P-O model, Šimleša et al., 2018). Such a model may provide a logical and straightforward vision of a complex process. Inputs are the conditions for the processes to start, what we can act upon. Core processes transform inputs into outputs. In a cognitive model, they correspond to individual intrapersonal unobservable mechanisms. An integrated cognitive-motivational model of ikigai refers to motivational and attentional functions as core processes. Finally, outputs are the observable and/or objective consequences we expect to achieve (e.g., behavior, psychological states), which also contribute to maintaining the system through a feedback loop.

**Figure 3:**
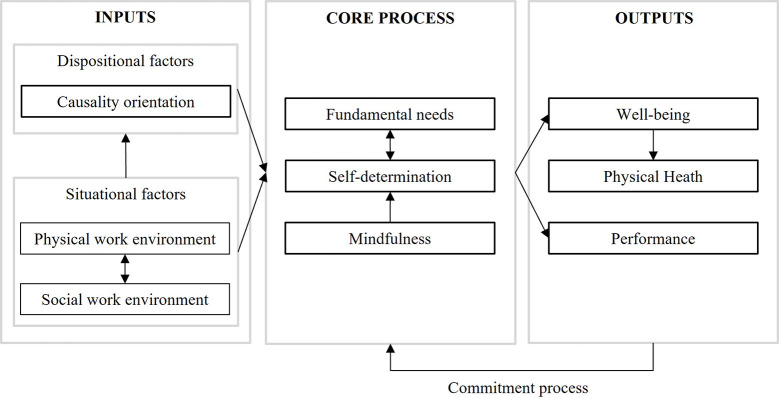
An Integrated Cognitive-Motivational Model of Ikigai

This model of ikigai is based on core processes composed of self-determination, fundamental needs, and mindfulness. We assume that these processes are triggered by two types of inputs: dispositional factors (causality orientation), and situational factors (social and physical work environment). Finally, outputs include well-being, physical health, and performance (see Figure 3). Ikigai process is self-nourished by a commitment feedback loop.

### Core Processes: Basic Needs, Self-Determination and Mindfulness

In Japanese literature, self-determination has already been included as a component of ikigai (Kumano, 2006), which directly refers to Self-Determination Theory (Deci & Ryan, 2000). Self-determination theory links human motivation to needs satisfaction, and includes three fundamental needs—need for competence, autonomy, and relatedness. Competence refers to the need to feel efficient and able to perform tasks at diverse levels of difficulty; autonomy corresponds to being at the root or source of one's activities; and relatedness refers to the need to feel associated to and supported by others. These higher-level goals refer to purposeful motivational strivings (Barrick et al., 2013) and meeting these needs results in psychological growth and well-being.

Self-determination theory mainly contrasts intrinsic and extrinsic motivation (Deci & Ryan, 1985, 2000, 2002), notably in a work context (Deci et al., 2017; Gagné & Deci, 2005). Intrinsic motivation refers to activities conducted for themselves, for pleasure, without external constraint (Deci & Ryan, 2000). An intrinsically motivated individual will perform activities for their own sake, without the need for reinforcement. Extrinsic motivation is characterized by reinforcements that are external to the individual (e.g., rewards).

Self-determination theory introduces variations of extrinsic motivation along a regulation continuum (Table 1) ranging from the least self-determined motivation (extrinsic) to the most self-determined one (intrinsic). In between, motivation is more or less internalized and characterized by five types of regulations (Deci & Ryan, 2000).

**Table 1:** The Self-Determination Continuum

Behavior	Non-determined	Self-determined				
Type of Motivation	Amotivation	Extrinsic motivation				Intrinsic motivation
Type of Regulation	Non- regulation	External regulation	Introjected regulation	Identified regulation	Integrated regulation	Intrinsic regulation
Locus of Causality	Impersonal	External	Somewhat external	Somewhat internal	Internal	Internal

*Note.* Taken from Deci & Ryan, 2000, p. 237.

Individuals expressing external regulation seek to achieve positive consequences which do not depend on themselves, such as obtaining rewards and avoiding negative consequences (e.g., punishment; Deci & Ryan, 2000). In introjected regulation, individuals seek to achieve internal positive consequences (e.g., self-esteem) and avoid negative consequences (e.g., feelings of guilt or shame). Identified regulation introduces the notion of values: individuals identify with the perceived value of a behavior. Identification contributes to the internalization of values, which generates commitment and performance (Deci & Ryan, 2000). Finally, integrated regulation “involves identifying with the importance of behaviors but also integrating those identifications with other aspects of the self” (Deci & Ryan, 2000, p. 236).

Self-determination theory seems to overlap in many ways with ikigai: Japanese literature mainly refers to intrinsic factors, but extrinsic factors are not excluded (Fukuzawa et al., 2019) and introjected motivation is mentioned through self-esteem (Kumano, 2006; Shirai et al., 2006). Identified and integrated regulation processes are also identifiable through the role of existential values for ikigai (Kamiya, 1966; Kumano, 2006).

Regarding fundamental needs, ikigai may be predominantly linked to the need for relatedness, which should generally be more salient in collectivist cultures. The social dimension of ikigai was also observed in empirical research (Fukuzawa et al., 2019). Furthermore, ikigai contributes to meeting needs for autonomy and competence through, for example, self-fulfillment, freedom of choice, and autonomous growth (Kamiya, 1966; Kumano, 2006).

Self-determination theory also fits to Winn's diagram of ikigai. Intrinsic regulation corresponds to “what I love”, extrinsic regulation to “what I am paid for”, introjected regulation to “what I am qualified for” and identified as well as integrated regulation to “what the world needs”. However, there may be variations in the relative importance of identified vs. intrinsic motivation between self-determination theory and ikigai. As self-determination theory is focused on personal growth, intrinsic motivation is considered as the ultimate achievement, whereas in ikigai philosophy, and consistent to its definition, meaningfulness, usefulness, or altruistic goals should be considered above intrinsic pleasure and satisfaction. The “what the world needs” area may bring most of the inspirational power of Winn's diagram, because this idea of achieving something greater than one’s own pleasure (self-transcendence) leads one to question the meaning of life in a deeper way.

More recent developments of self-determination theory account for this issue. For example, it was stressed that competitive individualism and capitalistic societies may hinder altruism and prosocial purposes, as well as lead to unsustainable attitudes and behaviors (Ryan et al., 2008). Self-determination alone cannot balance such sociocultural bias: the awareness of what is worth doing, the desire to make meaningful choices and the realignment to one’s values require the mindfulness process. Mindfulness is defined as “awareness of what is occurring in the present moment, and is characterized by an open and receptive processing of events, both internal and external” (Ryan et al., 2008, p. 158). Mindfulness is also central to eudaimonia or psychological well-being. Hence, we decided to include mindfulness as a core process of ikigai.

Mindfulness is defined as “the awareness that emerges through paying attention on purpose, in the present moment, and nonjudgmentally to the unfolding of experience moment by moment” (Kabat-Zinn, 2003, p. 145). It is a state of open and receptive awareness and processing of events. Contrary to flow, which is a narrow and internally-oriented attentional focus (Šimleša et al., 2018), mindfulness would rather correspond to a large and externally oriented attentional focus. Mindfulness emerges through intention, attention, and attitude (Ruedy & Schweitzer, 2010; Shapiro et al., 2006, 2008) and its benefits on mental and physical health are well documented (Hölzel et al., 2011; Kabat-Zinn, 1982): it promotes well-being (Birtwell et al., 2019; Walsh & Shapiro, 2006), improves performance and relationships (Schultz et al., 2015) including in professional contexts (Chiesa & Serretti, 2009; Lau et al., 2006). It also increases leadership skills (Brewer et al., 2011).

### Inputs

As the Japanese model of ikigai does little to integrate dispositional and situational factors, we investigated preconditions to self-determination and integrated them as ikigai potential drivers. We distinguish between situational and dispositional inputs to self-determination in the workplace (Barrick et al., 2013; Gagné & Deci, 2005): situational inputs can be found in physical and social environment (e.g., job content, job context, and work climate), and dispositional input correspond to individual differences (e.g., causality orientation, personality).

#### Situational Factors: Social and Physical Work Environment

According to the Cognitive Evaluation Theory, situational variables may impact motivation by affecting the feeling of autonomy and/or competence (Deci & Ryan, 1985; Gagné & Deci, 2005; Vallerand et al., 1987). The so-called “perceived locus of causality” ranges from internal (feeling of autonomy) to external (feeling controlled). External events can move the locus of causality: for example, a tangible reward can decrease the sense of freedom and intrinsic motivation while a merit reward can increase one’s feeling of competence and intrinsic motivation.

Situational factors include psychological and environmental working conditions, which impact job satisfaction (Pujol-Cols & Dabos, 2019). For example, job characteristics (skill variety, task identity and significance, autonomy, feedback) can foster intrinsic motivation (Hackman & Oldham, 1976; Piccolo & Colquitt, 2006).

Physical work environment is also considered as an input or a moderator to self-determination (Bamberger, 2008), as it impacts work satisfaction, performance (Bechtel, 2010; Chandrasekar, 2011), communication, collaboration (Brill & Weidemann, 2001), engagement, and employee morale (Chandrasekar, 2011). In line with the Theory of Purposeful Work Behavior, discordant work situations, inconsistencies or lack of compatibility with basic needs should be removed from work environment (Barrick et al., 2013) to prevent any detrimental effect on ikigai.

#### Dispositional Factors: Causality Orientation

Dispositional factors are relatively stable variables (Caspi et al., 2005; Dormann et al., 2006) that affect attitudes and behaviors at work (Judge et al., 2008; Ones et al., 2007; Pujol-Cols & Dabos, 2019) as well as work motivation (Austin & Klein, 1996). Consistently to the three-level hierarchical model of motivation (personality, life domain, and state motivation; Vallerand, 1997), Amabile et al. (1994) show that intrinsic-extrinsic motivational orientation is relatively stable across time and situations. The Causality Orientation Theory (Deci & Ryan, 1985) even considers this motivational orientation as a trait. Finally, dispositional and situational factors interact: autonomous causality orientation leads to intrinsic motivation disregarding situational factors (Gagné & Deci, 2005) and controlled orientation promoting extrinsic motivation is more strongly influenced by job characteristics.

### Outputs

The Japanese model of ikigai is more accurate on outputs, which is consistent to seeing ikigai as a state. On the basis of both ikigai and self-determination literature, we integrated three outputs: well-being, physical health, and performance.

#### Well-Being as a Psychological State

As ikigai can be viewed as a process and well-being is a state (Imai et al., 2009), the question of the relationship between ikigai and well-being is central to the Japanese literature (Fukuzawa et al., 2019; Iida & Oguma, 2013; Kumano, 2006; Shirai et al., 2006). Subjective or hedonic well-being relates to how people feel and think about their lives (Diener, 1984). It combines an affective dimension (high levels of positive affect and low levels of negative affect) and a cognitive dimension relying on global life satisfaction and on evaluation of specific life domains (e.g., job satisfaction or marital satisfaction). Psychological well-being, also called eudemonic well-being (Ryan & Deci, 2001), is another construct based on cognitive evaluations of long-term life experience such as autonomy, environmental mastery, personal growth, positive relations with others, purpose in life, and self-acceptance. The distinction between all these constructs (subjective, affective, cognitive and psychological well-being) is theoretical and conceptual, but empirical studies tend to show that all well-being dimensions are positively inter-correlated (Anglim et al., 2020). Furthermore, although life events impact subjective well-being, in particular in its cognitive dimension (Luhmann et al., 2012), well-being in general appears to be rather stable over time and related to personality profile for about half of its variance (Anglim et al., 2020). It is also interesting to note that the causal relation from subjective well-being to job satisfaction seems to be stronger than the causal relation from job satisfaction to subjective well-being (Bowling et al., 2010).

From a linguistic viewpoint, ikigai is closer to eudemonic well-being and “Shiawase” better corresponds to hedonic well-being. Although ikigai literature mentions all kinds of well-being (Fukuzawa et al., 2019; Shirai et al., 2006; Tanno et al., 2009), the Japanese ikigai model (Kumano, 2006) also emphasizes eudemonic well-being as more central than hedonic well-being. In our aim to formalize a testable model of ikigai in a professional context, we chose to include well-being through the PERMA framework, as it is a theory of the building blocks of well-being (Seligman, 2018) and may contribute thereby to understand ikigai dynamics more accurately. Those building blocks are: Positive emotions (feeling joyful), Engagement (interest and absorption in the task), positive Relationships (satisfaction with one's social relationships), Meaning (the belief that one’s life is valuable and connected to something greater), and Accomplishment (making progress, experiencing self-esteem and sense of achievement). PERMA components have been independently validated as contributing to overall well-being (Kern et al., 2015; Seligman, 2011). The Japanese model of ikigai (Kumano, 2006) includes at least four PERMA components: positive affects (P), positive relations (R), meaning of life (M) and sense of fulfillment (A). They are all positioned from the second to the third peripheral level of ikigai, which is consistent with their output status in our model.

PERMA appears as a consistent output to our core processes, as self-determined, intrinsic levels of motivation directly generate pleasure (P), engagement (E) and accomplishment (A). Besides, positive relations (R) and accomplishment (A) correspond to fundamental needs (relatedness and competence) motivating self-determination process and meaning (M) may result from the mindfulness process.

#### Physical Health

Consistent to the Japanese model which integrates it peripherally, we consider physical health as an output. Finding and experiencing ikigai is frequently associated to better physical health (Kotera et al., 2021), and a weak ikigai is associated to “poor general health” (Nakanishi, 1999). In particular, ikigai reduces risks of diseases (Sone et al., 2008; Tanno et al., 2009). Furthermore, research also addresses the link between well-being and physical health—in particular subjective well-being (Diener & Chan, 2011). Engagement (E) and meaning (M) could play an important role in this link (Roepke et al., 2014).

#### Performance

Although ikigai literature does not explicitly refer to performance, we added this output for two main reasons. Firstly, our aim to model ikigai in the workplace calls for further examination of the effects of ikigai on performance. Secondly, performance is considered as a direct output of intrinsic motivation (Vroom, 1994), whereas extrinsic motivation can degrade performance (Gagné & Deci, 2005).

### Feedback Loop: Commitment Process

Commitment is the process linking behaviors (i.e., observable outputs) to the individual's attitudes and cognitive processes (Kiesler & Sakumura, 1966). Commitment contributes to determining people's behaviors through the actions they have previously taken and not only through their opinions and values. From the Japanese model of ikigai, we introduced commitment as a feedback loop to nurture a lifelong, self-maintained ikigai process.

## Conclusion and Future Orientations

This integrated cognitive-motivational model of ikigai may bring several contributions. The first one is to gather Japanese literature and contemporary psychological literature to build a unified consistent model. In this respect, we found self-determination combined with mindfulness as the most likely core process of ikigai. This enabled us to identify plausible dispositional and situational factors likely to enhance ikigai or explain individual differences in ikigai. Considering ikigai in the workplace, we also introduced performance as an output variable resulting from ikigai and consistent with known outcomes of self-determination and mindfulness. With regard to current approach of well-being and performance at work, the effort to conceptualize ikigai led us to introduce mindfulness in the core processes as a way to highlight the importance of meaningfulness at work and eudemonic well-being, beyond individual pleasure and hedonic well-being.

Our model may nonetheless hold several limitations. The first one is its potential cultural bias challenging the possibility to adopt a Japanese way of living in a European or North American work context. Ikigai questions our self-perception (Mathews, 1996). However, because we managed to account for most of ikigai features through existing psychological theories, we still feel confident in the relevance of our model to inspire new ways of shaping the workplace. Secondly, using ikigai in the workplace can be viewed as a misappropriation of the concept. The attempt to translate Winn's diagram in theoretical terms led us to introduce extrinsic rewards into the ikigai process. Research repeatedly highlighted the detrimental effects of extrinsic motivation on performance (Amabile, 1983). In this respect, we believe that the success of ikigai process to generate eudemonic well-being, physical health and performance will depend on the respective salience of internal and extrinsic motivators for each individual and each situation—which further emphasizes the importance of dispositional and situational factors.

The integrated cognitive-motivational model of ikigai remains to be empirically tested. As research on ikigai has been conducted mainly with students and elderly people (Kumano, 2018), this would require to study ikigai within working population. Furthermore, we can study the extent to which variations in inputs (dispositional and situational factors) impact ikigai core processes and outputs. We can also conduct field interventions to influence situational factors and monitor their effects on ikigai. We can imagine introducing new managerial practices and new working conditions likely to foster intrinsic regulation, mindfulness, self-determination, and subsequently ikigai. We also intend to use our model as a framework to design tools (e.g., digital or robotic tools) that would be specified not only to meet functional needs (e.g., productivity, profitability), but also to meet workers' motivational needs (i.e., needs for competence, autonomy and relatedness), increase their sense of purpose, their well-being at work and more generally their feeling of a life worth living.

## References

[r1] Amabile, T. M. (1983). The social psychology of creativity: A componential conceptualization. Journal of Personality and Social Psychology, 45(2), 357–376. 10.1037/0022-3514.45.2.357

[r2] Amabile, T. M., Hill, K. G., Hennessey, B. A., & Tighe, E. M. (1994). The Work Preference Inventory: Assessing intrinsic and extrinsic motivational orientations. Journal of Personality and Social Psychology, 66(5), 950–967. 10.1037/0022-3514.66.5.950 8014837

[r3] Anglim, J., Horwood, S., Smillie, L. D., Marrero, R. J., & Wood, J. K. (2020). Predicting psychological and subjective well-being from personality: A meta-analysis. Psychological Bulletin, 146(4), 279–323. 10.1037/bul0000226 31944795

[r4] Austin, J. T., & Klein, H. J. (1996). Work motivation and goal striving. In K. R. Murphy (Ed.), *Individual differences and behavior in organizations*, pp. 209–257). Jossey-Bass.

[r5] Bamberger, P. (2008). Beyond contextualization: Using context theories to narrow the micro-macro gap in management research. Academy of Management Journal, 51(5), 839–846. 10.5465/amj.2008.34789630

[r6] Barrick, M. R., Mount, M. K., & Li, N. (2013). The theory of purposeful work behavior: The role of personality, job characteristics, and experienced meaningfulness. Academy of Management Review, 38(1), 132–153. 10.5465/amr.2010.0479

[r7] Bechtel, R. B. (2010). *Environmental psychology*. Wiley.

[r8] Birtwell, K., Williams, K., van Marwijk, H., Armitage, C. J., & Sheffield, D. (2019). An exploration of formal and informal mindfulness practice and associations with wellbeing. Mindfulness, 10(1), 89–99. 10.1007/s12671-018-0951-y 30662573 PMC6320743

[r9] Bowling, N. A., Eschleman, K. J., & Wang, Q. (2010). A meta‐analytic examination of the relationship between job satisfaction and subjective well‐being. Journal of Occupational and Organizational Psychology, 83(4), 915–934. 10.1348/096317909X478557

[r10] Brewer, J. A., Worhunsky, P. D., Gray, J. R., Tang, Y.-Y., Weber, J., & Kober, H. (2011). Meditation experience is associated with differences in default mode network activity and connectivity. Proceedings of the National Academy of Sciences of the United States of America, 108(50), 20254–20259. 10.1073/pnas.1112029108 22114193 PMC3250176

[r11] Brill, M., & Weidemann, S. (2001). *Disproving widespread myths about workplace design.* Kimball International.

[r12] Buettner, D. (2009). *How to live 100+*. TED Ideas Worth Spreading. https://www.ted.com/talks/dan_buettner_how_to_live_to_be_100

[r13] Caspi, A., Roberts, B., & Shiner, R. (2005). Personality development: Stability and change. Annual Review of Psychology, 56, 453–484. 10.1146/annurev.psych.55.090902.141913 15709943

[r14] Chandrasekar, K. (2011). Workplace environment and its impact on organisational performance in public sector organisations. International Journal of Enterprise Computing and Business Systems, 1(1), 1–19.

[r15] Chiesa, A., & Serretti, A. (2009). Mindfulness-based stress reduction for stress management in healthy people: A review and meta-analysis. Journal of Alternative and Complementary Medicine, 15(5), 593–600. 10.1089/acm.2008.0495 19432513

[r16] Deci, E. L., Olafsen, A. H., & Ryan, R. M. (2017). Self-determination theory in work organizations: The state of a science. Annual Review of Organizational Psychology and Organizational Behavior, 4, 19–43. 10.1146/annurev-orgpsych-032516-113108

[r17] Deci, E. L., & Ryan, R. M. (1985). *Intrinsic Motivation and self-determination in human behavior*. Springer US. 10.1007/978-1-4899-2271-7

[r18] Deci, E. L., & Ryan, R. M. (2000). The “what” and “why” of goal pursuits: Human needs and the self-determination of behavior. Psychological Inquiry, 11(4), 227–268. 10.1207/S15327965PLI1104_01

[r19] Deci, E. L., & Ryan, R. M. (2002). *Handbook of self-determination research*. University of Rochester Press.

[r20] Diener, E. (1984). Subjective well-being. Psychological Bulletin, 95(3), 542–575. 10.1037/0033-2909.95.3.542 6399758

[r21] Diener, E., & Chan, M. Y. (2011). Happy people live longer: Subjective well‐being contributes to health and longevity. Applied Psychology. Health and Well-Being, 3(1), 1–43. 10.1111/j.1758-0854.2010.01045.x26286968

[r22] Dormann, C., Fay, D., Zapf, D., & Frese, M. (2006). A state-trait analysis of job satisfaction: On the effect of core self-evaluations. Applied Psychology, 55(1), 27–51. 10.1111/j.1464-0597.2006.00227.x

[r23] Fido, D., Kotera, Y., & Asano, K. (2020). English translation and validation of the Ikigai-9 in a UK sample. International Journal of Mental Health and Addiction, 18, 1352–1359. 10.1007/s11469-019-00150-w

[r24] Fukuzawa, A., Katagiri, K., Harada, K., Masumoto, K., Chogahara, M., Kondo, N., & Okada, S. (2019). A longitudinal study of the moderating effects of social capital on the relationships between changes in human capital and ikigai among Japanese older adults. Asian Journal of Social Psychology, 22(2), 172–182. 10.1111/ajsp.12353

[r25] Gagné, M., & Deci, E. (2005). Self-determination theory and work motivation. Journal of Organizational Behavior, 26, 331–362. 10.1002/job.322

[r26] Hackman, J. R., & Oldham, G. R. (1976). Motivation through the design of work: Test of a theory. Organizational Behavior and Human Performance, 16(2), 250–279. 10.1016/0030-5073(76)90016-7

[r27] Hikmawan, R., Sari, D. P., Majid, N. A., Ridwan, T., Nuriyah, W., Aprilia, L., & Diani, D. (2019). Development of ikigai instructional method to cultivate computational thinking of millennial generations. Journal of Physics: Conference Series, 1318, 012007. 10.1088/1742-6596/1318/1/012007

[r28] Hofstede, G., Hofstede, G. J., & Minkov, M. (2010). *Cultures and organizations: Software of the mind*. McGraw-Hill.

[r29] Hölzel, B. K., Carmody, J., Vangel, M., Congleton, C., Yerramsetti, S. M., Gard, T., & Lazar, S. W. (2011). Mindfulness practice leads to increases in regional brain gray matter density. Psychiatry Research: Neuroimaging, 191(1), 36–43. 10.1016/j.pscychresns.2010.08.006 21071182 PMC3004979

[r30] Iida, K., & Oguma, Y. (2013). Relationships between flow experience, ikigai, and sense of coherence in tai chi practitioners. Holistic Nursing Practice, 27(5), 260–267. 10.1097/HNP.0b013e31829b9199 23925345

[r31] Imai, T. (2012). The reliability and validity of a new scale for measuring the concept of Ikigai (Ikigai-9). Japanese Journal of Public Health, 59(7), 7. 10.1097/HNP.0b013e31829b9199 22991767

[r32] Imai, T., Osada, H., & Nishimura, Y. (2009). The structure of ikigai concept for retirees over 60 years old, The difference between ikigai and subjective well-being. Japanese Journal of Geriatric Psychiatry, 31(3), 366–377.

[r33] Ishida, R. (2012). Decreasing anxiety in stutterers through the association between “purpose in life/ikigai” and emotions. Global Journal of Health Science, 4(5), 120–124. 10.5539/gjhs.v4n5p120 22980384 PMC4776915

[r34] Judge, T., Klinger, R., Simon, L., & Yang, I. (2008). The contributions of personality to organizational behavior and psychology: Findings, criticisms, and future research directions. Social and Personality Psychology Compass, 2(5), 1982–2000. 10.1111/j.1751-9004.2008.00136.x

[r35] Kabat-Zinn, J. (1982). An outpatient program in behavioral medicine for chronic pain patients based on the practice of mindfulness meditation: Theoretical considerations and preliminary results. General Hospital Psychiatry, 4(1), 33–47. 10.1016/0163-8343(82)90026-3 7042457

[r36] Kabat-Zinn, J. (2003). Mindfulness-based interventions in context: Past, present, and future. Clinical Psychology: Science and Practice, 10(2), 144–156. 10.1093/clipsy.bpg016

[r37] Kamiya, M. (1966). *Ikigai ni tsuite*. Misuzu Shobō.

[r38] Kern, M. L., Waters, L. E., Adler, A., & White, M. A. (2015). A multidimensional approach to measuring well-being in students: Application of the PERMA framework. Journal of Positive Psychology, 10(3), 262–271. 10.1080/17439760.2014.936962 25745508 PMC4337659

[r39] Kiesler, C. A., & Sakumura, J. (1966). A test of a model for commitment. Journal of Personality and Social Psychology, 3(3), 349–353. 10.1037/h0022943 5906339

[r40] Kono, S., Walker, G. J., Ito, E., & Hagi, Y. (2019). Theorizing leisure’s roles in the pursuit of ikigai (life worthiness): A mixed-methods approach. Leisure Sciences, 41(4), 237–259. 10.1080/01490400.2017.1356255

[r41] Kotera, Y., Kaluzeviciute, G., Gulcan, G., McEwan, K., & Chamberlain, K. (2021). Health benefits of ikigai: A review of literature. In Y. Kotera & D. Fido (Eds.), *Ikigai: Towards a psychological understanding of a life worth living* (pp. 1–13). Concurrent Disorders Society Publishing.

[r42] Kumano, M. (2003). Two-dimensional model of “ikigai” based on profiles of the view of life. Japanese Journal of Health Psychology, 16(2), 68–76. 10.11560/jahp.16.2_68

[r43] Kumano, M. (2006). The structure of ikigai and similar concepts. Japanese Journal of Health Psychology, 19(1), 56–66. 10.11560/jahp.19.1_56

[r44] Kumano, M. (2012). *Ikigai-keisei-no-shinrigaku* [A psychology of ikigai development]. Kazama Shyobou.

[r45] Kumano, M. (2013). Ikigai-keisei-moderu-no-sokutei-shyakudo-no-sakusei: Ikigai-purosesu-shyakudo-to-ikigai-jyoutai-shyakudo [Construction of scales for the ikigai development model: The scales for ikigai processes and ikigai states]. Bulletin of Education, 39, 1–11.

[r46] Kumano, M. (2018). On the concept of well-being in Japan: Feeling shiawase as hedonic well-being and feeling ikigai as eudaimonic well-being. Applied Research in Quality of Life, 13(2), 419–433. 10.1007/s11482-017-9532-9

[r47] Lau, M. A., Bishop, S. R., Segal, Z. V., Buis, T., Anderson, N. D., Carlson, L., Shapiro, S., Carmody, J., Abbey, S., & Devins, G. (2006). The Toronto Mindfulness Scale: Development and validation. Journal of Clinical Psychology, 62(12), 1445–1467. 10.1002/jclp.20326 17019673

[r48] Luhmann, M., Hofmann, W., Eid, M., & Lucas, R. E. (2012). Subjective well-being and adaptation to life events: A meta-analysis. Journal of Personality and Social Psychology, 102(3), 592–615. 10.1037/a0025948 22059843 PMC3289759

[r49] Markus, H., & Kitayama, S. (1991). Culture and the self: Implications for cognition, emotion, and motivation. Psychological Review, 98, 224–253. 10.1037/0033-295X.98.2.224

[r50] Mathews, G. (1996). The stuff of dreams, fading: Ikigai and “the Japanese self”. Ethos, 24(4), 718–747. 10.1525/eth.1996.24.4.02a00060

[r51] Mori, K., Kaiho, Y., Tomata, Y., Narita, M., Tanji, F., Sugiyama, K., Sugawara, Y., & Tsuji, I. (2017). Corrigendum to “Sense of life worth living (ikigai) and incident functional disability in elderly Japanese: The Tsurugaya Project”. Journal of Psychosomatic Research, 96, 106–106. 10.1016/j.jpsychores.2017.03.006 28314550

[r52] Murray, D. W. (1993). What is the Western concept of the self? On forgetting David Hume. Ethos, 21(1), 3–23. 10.1525/eth.1993.21.1.02a00010

[r53] Nakanishi, N. (1999). “Ikigai” in older Japanese people. Age and Ageing, 28(3), 323–324. 10.1093/ageing/28.3.323 10475874

[r54] Ones, D., Dilchert, S., Viswesvaran, C., & Judge, T. (2007). In support of personality assessment in organizational settings. Personnel Psychology, 60(4), 995–1027. 10.1111/j.1744-6570.2007.00099.x

[r55] Oyserman, D., & Lee, S. W. S. (2008). Does culture influence what and how we think? Effects of priming individualism and collectivism. Psychological Bulletin, 134(2), 311–342. 10.1037/0033-2909.134.2.311 18298274

[r56] Piccolo, R. F., & Colquitt, J. A. (2006). Transformational leadership and job behaviors: The mediating role of core job characteristics. Academy of Management Journal, 49(2), 327–340. 10.5465/amj.2006.20786079

[r57] Pujol-Cols, L., & Dabos, G. (2019). Dispositional and situational factors at work: A validation of scales and examination of effects on job satisfaction. Academia Revista Latinoamerica de Administracion, 33(1), 49–70. 10.1108/ARLA-12-2017-0355

[r58] Roepke, A. M., Jayawickreme, E., & Riffle, O. M. (2014). Meaning and health: A systematic review. Applied Research in Quality of Life, 9(4), 1055–1079. 10.1007/s11482-013-9288-9

[r59] Ruedy, N. E., & Schweitzer, M. E. (2010). In the moment: The effect of mindfulness on ethical decision making. Journal of Business Ethics, 95, 73–87. 10.1007/s10551-011-0796-y

[r60] Ryan, R. M., & Deci, E. L. (2000). Self-determination theory and the facilitation of intrinsic motivation, social development, and well-being. American Psychologist, 55(1), 68–78. 10.1037/0003-066X.55.1.68 11392867

[r62] Ryan, R. M., & Deci, E. L. (2001). On happiness and human potentials: A review of research on hedonic and eudaimonic well-being. Annual Review of Psychology, 52, 141–166. 10.1146/annurev.psych.52.1.141 11148302

[r61] Ryan, R., Huta, V., & Deci, E. (2008). Living well: A self-determination theory perspective on eudaimonia. Journal of Happiness Studies, 9, 139–170. 10.1007/s10902-006-9023-4

[r63] Schultz, P. P., Ryan, R. M., Niemiec, C. P., Legate, N., & Williams, G. C. (2015). Mindfulness, work climate, and psychological need satisfaction in employee well-being. Mindfulness, 6(5), 971–985. 10.1007/s12671-014-0338-7

[r64] Seligman, M. (2011). *Flourish: A visionary new understanding of happiness and well-being.* Free Press.

[r65] Seligman, M. (2018). PERMA and the building blocks of well-being. Journal of Positive Psychology, 13(4), 333–335. 10.1080/17439760.2018.1437466

[r66] Shapiro, S. L., Carlson, L. E., Astin, J. A., & Freedman, B. (2006). Mechanisms of mindfulness. Journal of Clinical Psychology, 62(3), 373–386. 10.1002/jclp.20237 16385481

[r67] Shapiro, S. L., Oman, D., Thoresen, C. E., Plante, T. G., & Flinders, T. (2008). Cultivating mindfulness: Effects on well‐being. Journal of Clinical Psychology, 64(7), 840–862. 10.1002/jclp.20491 18484600

[r68] Shirai, K., Iso, H., Fukuda, H., Toyoda, Y., Takatorige, T., & Tatara, K. (2006). Factors associated with “ikigai” among members of a public temporary employment agency for seniors (Silver Human Resources Centre) in Japan: Gender differences. Health and Quality of Life Outcomes, 4(1), 12. 10.1186/1477-7525-4-12 16504162 PMC1450260

[r69] Šimleša, M., Guegan, J., Blanchard, E., Tarpin-Bernard, F., & Buisine, S. (2018). The flow engine framework: A cognitive model of optimal human experience. Europe’s Journal of Psychology, 14(1), 232–253. 10.5964/ejop.v14i1.1370 29899807 PMC5973526

[r70] Singelis, T. M., Triandis, H. C., Bhawuk, D. P., & Gelfand, M. J. (1995). Horizontal and vertical dimensions of individualism and collectivism: A theoretical and measurement refinement. Cross-Cultural Research, 29(3), 240–275. 10.1177/106939719502900302

[r71] Smith, R. J. (1991). *Memory and time in the formation of the not entirely sociocentric self* [Keynote address]. Conference on the self and the social order in China, India, and Japan, East-West Center, Honolulu, HI, United States of America.

[r72] Sone, T., Nakaya, N., Ohmori, K., Shimazu, T., Higashiguchi, M., Kakizaki, M., Kikuchi, N., Kuriyama, S., & Tsuji, I. (2008). Sense of life worth living (ikigai) and mortality in Japan: Ohsaki Study. Psychosomatic Medicine, 70(6), 709–715. 10.1097/PSY.0b013e31817e7e64 18596247

[r73] Spiro, M. E. (1993). Is the Western conception of the self “peculiar” within the context of the world cultures? Ethos, 21(2), 107–153. 10.1525/eth.1993.21.2.02a00010

[r74] Tanno, K., Sakata, K., Ohsawa, M., Onoda, T., Itai, K., Yaegashi, Y., & Tamakoshi, A. (2009). Associations of ikigai as a positive psychological factor with all-cause mortality and cause-specific mortality among middle-aged and elderly Japanese people: Findings from the Japan Collaborative Cohort Study. Journal of Psychosomatic Research, 67(1), 67–75. 10.1016/j.jpsychores.2008.10.018 19539820

[r75] Vallerand, R. J. (1997). Toward a hierarchical model of intrinsic and extrinsic motivation. Advances in Experimental Social Psychology, 29, 271–360. 10.1016/S0065-2601(08)60019-2

[r76] Vallerand, R. J., Blais, M. R., Lacouture, Y., & Deci, E. L. (1987). L’Échelle des Orientations Générales à la Causalité : Validation canadienne française du General Causality Orientations Scale [The General Causality Orientations Scale: The Canadian French version of the General Causality Orientations Scale]. Canadian Journal of Behavioural Science/Revue Canadienne des Sciences du Comportement*, *19(1), 1–15. 10.1037/h0079872

[r77] Vandroux, R., & Auzoult-Chagnault, L. (in press). Validation francophone de l’échelle Ikigai-9 [Validation of the Ikigai-9 scale in French]. Psychologie Française. Advance online publication. 10.1016/j.psfr.2022.12.001

[r78] Vroom, V. H. (1994). *Work and motivation*. Wiley.

[r79] Walsh, R., & Shapiro, S. L. (2006). The meeting of meditative disciplines and Western psychology: A mutually enriching dialogue. American Psychologist, 61(3), 227–239. 10.1037/0003-066X.61.3.227 16594839

[r80] Weiss, R. S., Bass, S. A., Heimovitz, H. K., & Oka, M. (2005). Japan’s Silver Human Resource Centers and participant well-being. Journal of Cross-Cultural Gerontology, 20(1), 47–66. 10.1007/s10823-005-3797-4 15870967

[r81] Winn, M. (2014, May 14). *What is your ikigai?* The View Inside. https://theviewinside.me/what-is-your-ikigai/

[r82] Zuzunaga, A. (2012). *Propósito*. Cosmograma. https://www.cosmograma.com/proposito.php

